# Gastroesophageal Reflux Disease in Obesity: Bariatric Surgery as Both the Cause and the Cure in the Morbidly Obese Population

**DOI:** 10.3390/jcm12175543

**Published:** 2023-08-25

**Authors:** Muaaz Masood, Donald Low, Shanley B. Deal, Richard A. Kozarek

**Affiliations:** 1 Division of Gastroenterology and Hepatology, Center for Digestive Health, Virginia Mason Franciscan Health, Seattle, WA 98101, USA; 2 Division of Thoracic Surgery, Center for Digestive Health, Virginia Mason Franciscan Health, Seattle, WA 98101, USA; 3 Division of General Surgery, Center for Weight Management, Virginia Mason Franciscan Health, Seattle, WA 98101, USA; 4 Center for Interventional Immunology, Benaroya Research Institute, Virginia Mason Franciscan Health, Seattle, WA 98101, USA

**Keywords:** gastroesophageal, reflux, GERD, esophagitis, morbid, obesity, bariatric, Roux-en-Y, gastric, bypass, surgery, sleeve, gastrectomy

## Abstract

Gastrointestinal reflux disease (GERD) is a chronic, highly prevalent condition in the United States. GERD can significantly impact quality of life and lead to complications including aspiration pneumonia, esophageal stricture, Barrett’s esophagus (BE) and esophageal cancer. Obesity is a risk factor for GERD, which often improves with weight loss and bariatric surgery. Though the incidence of bariatric surgery, in particular, minimally invasive sleeve gastrectomy, has risen in recent years, emerging data has revealed that the severity or new onset of GERD may follow bariatric surgery. We performed a literature review to provide a detailed analysis of GERD with an emphasis on bariatric surgery as both the cure and the cause for GERD in the morbidly obese population. We also describe the pathophysiological mechanisms, management approach and treatment strategies of GERD following bariatric surgery.

## 1. Introduction

It is reported that two out of five individuals have experienced reflux symptoms in the past and approximately one-third of individuals reported reflux symptoms within the past week, making GERD a common chronic condition in the United States [1]. The prevalence in the general population is estimated to be 18.1–27.8% [2]. GERD was the most frequent gastrointestinal diagnosis listed in outpatient clinic visits in the United States in 2009 [3].

The condition typically manifests as heartburn and regurgitation. Atypical or extraesophageal symptoms may be present in up to one-third of patients [4]. These include cough, laryngitis, asthma and chest pain [5]. GERD has been further classified as non-erosive reflux disease (NERD) and erosive reflux disease (ERD) based on endoscopic findings, and both can significantly impact quality of life and lead to complications, including Barrett’s esophagus and esophageal cancer [6,7].

Obesity is a risk factor for GERD and improves with weight loss [8,9]. Interestingly, as the incidence of bariatric surgery, particularly minimally invasive sleeve gastrectomy, has risen in recent years, emerging data has revealed that GERD may worsen after certain bariatric surgeries [10,11,12,13,14,15]. New-onset GERD can also develop post-bariatric surgery [12,13,16,17,18]. We conducted a literature review to provide a detailed analysis of GERD with an emphasis on bariatric surgery as both the cure and the cause in the morbidly obese population. We review the pathophysiological mechanisms, management approach and treatment strategies of GERD following bariatric surgery.

## 2. Methods

We performed a narrative review of the literature to provide a detailed analysis of GERD following bariatric surgery using PubMed computerized search and Google Scholar for articles with the title or keywords “GERD”, “gastroesophageal”, “reflux”, “obesity”, “bariatric surgery”, “Roux-en-Y gastric bypass”, “laparoscopic”, “sleeve gastrectomy” and “gastric sleeve”. Systematic reviews, literature reviews, meta-analyses, randomized controlled trials and clinical trials were prioritized. Articles that were not available in English or full text were excluded. Duplicate articles were removed. Articles regarding reflux in the context of pregnancy were excluded. The articles were independently reviewed by all authors.

## 3. Discussion

### 3.1. Obesity as the Cause of GERD

Obesity, defined as a body-mass index (BMI) of ≥30 kg/m^2^, has substantially increased over the past several decades in the United States with an estimated prevalence of 41.9% [19,20]. Obesity is a well-established risk factor for GERD independent of diet and a concomitant rise in the incidence of GERD has been noted [8,21,22,23]. A meta-analysis of nine studies by Hampel et al. revealed a pooled adjusted odds ratios of 1.43 for GERD symptoms for BMI of 25–30 kg/m^2^ and 1.94 for BMI > 30 kg/m^2^, highlighting the dose–response relationship between obesity and reflux [9]. The relationship between obesity and GERD has also been noted in other parts of the world, including Europe and Asia, where rates of obesity are overall lower than the Western hemisphere, but have been rising in recent years [2,24,25,26,27,28]. Additionally, there has been an increase in the complications of GERD, including Barrett’s esophagus and esophageal adenocarcinoma [29]. Central obesity, in particular, has been associated with an increase in the risk of Barrett’s esophagus (BE) which may be in part due to reflux [30]. Some studies have suggested a role for adiponectin and leptin from adipocytes in mediating obesity and BE [31,32].

There are multiple mechanisms that predispose obese patients to reflux (Table 1). Transient lower esophageal sphincter relaxations (TLESRs) consist of spontaneous relaxations of the lower esophageal sphincter and crural diaphragm, which are not preceded by swallowing. The most common stimulus for TLESRs has been suggested to be distension of the proximal stomach. Obese patients have a significantly increased number of total TLESRs, and thus reflux and acid exposure, especially in the postprandial phase [33,34]. In a prospective study of 84 patients, TLESRs were demonstrated to correlate with BMI and waist circumference [35]. Similarly, Corley et al. demonstrated a strong association between abdominal diameter and reflux symptoms, especially in Caucasians in a large, cross-sectional study [36]. The link between BMI and reflux symptoms seems to be partially mediated by abdominal diameter. A recent, prospective study of 771 patients proposed that the waist-to-hip ratio may be a better predictor of esophageal acid exposure compared to BMI [37].

Obesity has been associated with an increased intra-abdominal pressure which may contribute to reflux of gastric contents into the esophagus [36]. Increased intra-abdominal pressure in obesity may result in the cephalad movement of the hiatal hernia and lead to reflux [38]. Pandolfino et al. explored the relationship between obesity and the esophagogastric junction pressure segment using high-resolution esophageal manometry in 285 patients [39]. The study concluded that obese patients were more likely to have an increased intragastric pressure, an augmented gastroesophageal pressure gradient, and disruption of esophagogastric junction, all of which can predispose to reflux. These findings were also correlated with waist circumference [39].

The gastroesophageal flap valve plays an important role in preventing the reflux of gastric contents into the esophagus. There is a high prevalence of hiatal hernias in obese patients, which leads to functional impairment of the esophagogastric junction [40]. Hiatal hernia involves herniation of the stomach through the esophageal hiatus into the mediastinum. Obesity has been linked to an increased separation between the lower esophageal sphincter and the crural diaphragm, which may predispose to the formation of a hiatal hernia [38]. In a study of 345 patients scheduled to undergo bariatric surgery, Suter et al. discovered a hiatal hernia on endoscopy in over 50% of patients [41]. More recently, Yen et al. found a hiatal hernia in 33% of patients with morbid obesity using high-resolution impedance manometry, and all patients with hiatal hernia had erosive esophagitis on endoscopy [42]. GERD is associated with a low esophageal sphincter pressure in patients with hiatal hernia, and hiatal hernias may also be larger in size in this population [43]. Of note, the baseline pressure of the lower esophageal sphincter, gastric volume, gastric acid production and gastric emptying are likely normal in obese patients, though the data are limited [44,45,46]. Gastric emptying may be delayed after a large, high-calorie meal which would increase risk of reflux [38]. The compromise in the esophagogastric junction and diaphragmatic hiatus coupled with a rise in intragastric pressure from abdominal obesity all provide the ideal conditions for the development of reflux.

### 3.2. Bariatric Surgery as the Treatment of GERD

**Figure 1:**
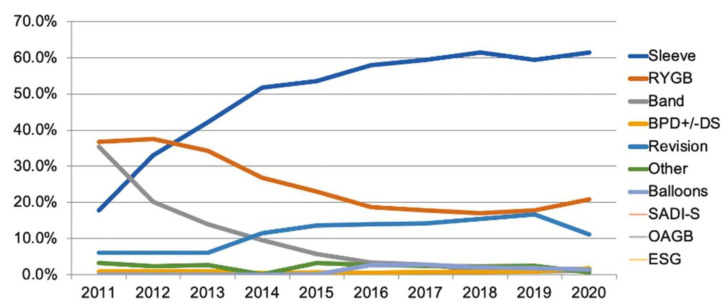
Trends in metabolic and bariatric surgery are shown from 2012 to 2020. BPD +/− DS = biliopancreatic diversion +/− duodenal switch; ESG = endoscopic sleeve gastroplasty; OAGB = one-anastomosis gastric bypass; RYGB = Roux-en-Y gastric bypass; SADI-S = single-anastomosis duodeno-ileostomy with sleeve [10].

Bariatric surgery, specifically Roux-en-Y gastric bypass (RYGB), is highly effective for the treatment of GERD. The majority of bariatric surgeries are performed in BMIs of 35–55. GERD has been reported in as many as 62.4% to 73% of bariatric surgery candidates [47,48]. There has been an overall gradual increase in bariatric surgeries over the last two decades, with an increase from 43.5 per 100,000 to 70.6 per 100,000 from 2006 to 2009 and a subsequent plateau in bariatric surgeries from 2010 to 2015 [49]. Recent trends in bariatric surgeries performed are shown in Figure 1. There was a reduction in the overall volume of metabolic and bariatric surgeries that coincided with the COVID-19 pandemic due to the suspension of elective surgeries [10]. RYGB and sleeve gastrectomy (SG) are the most commonly performed bariatric surgeries [10,49]. There has been a significant increase in SG in recent years and it is the most widely performed bariatric surgery. In parallel, there was a steady decline in RYGB procedures at the end of 2012 and beginning of 2013, and a plateau in 2017, with an upward trend in 2019 and 2020. There has also been a large increase in minimally invasive bariatric surgeries compared to open bariatric surgeries [49].

High rates of GERD improvement have been observed following RYGB (Table 2). A randomized controlled trial of 217 patients with a 5-year follow-up period revealed that remission of reflux was observed in 60.4% of cases after RYGB compared to 25% of cases after SG [50]. There was no significant difference in weight loss between the two groups in the trial [50]. Frezza et al. revealed a significant reduction in reflux symptoms following RYGB in a study of 152 patients [51]. Similarly, Perry et al. demonstrated improvement or resolution of GERD in all 57 patients postoperatively following RYGB [52]. Laparoscopic RYGB (LRYGB) has been shown to be superior to laparoscopic SG (LSG) in improving reflux (*p*-value = 0.003, odd ratio, 3.16, 95% CI, 1.48–6.76) [53]. The superior improvement in GERD with RYGB may be related to higher weight loss achieved with RYGB. Additionally, pathophysiologic differences, including a major reduction in the mass of parietal cells responsible for gastric acid secretion with RYGB, and mechanical differences between the two procedures also contribute to decreased risk of reflux with RYGB compared to SG [54,55]. It is important to note that, due to the increased prevalence of reflux in patients with hiatal hernias, concomitant hiatal hernia should be repaired at the time of bariatric surgery, as it has been shown to be safe [56,57].

### 3.3. Bariatric Surgery as the Cause of GERD: A Burning Issue

As the incidence of bariatric surgery rises, the persistent and de novo reflux following bariatric surgery has become a topic of concern. There has been mounting evidence that bariatric surgery, in particular SG, may lead to worsening of GERD (Table 3). According to a meta-analysis which compared the outcomes of SG and RYGB, the odds ratio for GERD after SG is five times higher (*p* < 0.001) [11]. In a retrospective review of 4832 patients, 84.1% of patients who underwent LSG continued to have GERD symptoms postoperatively, and 8.6% developed GERD postoperatively [16]. Preoperative GERD was associated with worse outcomes in the study [16]. Similarly, Sheppard et al. retrospectively reviewed 387 cases and noted increased PPI usage in patients after LSG in comparison to LRYGB [12]. Carter et al. concluded that LSG was associated with persistent reflux symptoms in patients who had preoperative GERD and increased risk of postoperative reflux symptoms in those who did not have these symptoms preoperatively [17]. Additional studies by Tai et al., Howard et al. and Matar et al. have also confirmed GERD following LSG [13,14,15].

Additionally, de novo GERD has also been documented following SG [12,13,16,17,18] (Table 4). A systematic review of 20 articles demonstrated that 8 out of 10 studies revealed rates of new-onset GERD following LSG [58,59]. The Swiss Multicenter Bypass or Sleeve Study (SM-BOSS) Trial of 217 patients reported de novo GERD in 31.6% of LSG patients vs. 10.7% in RYGB patients (*p* = 0.01) [50]. In a study of 110 patients who underwent LSG, the incidence of reflux symptoms and PPI intake were significantly increased compared to pre-operative values (68.1% vs. 33.6%, *p* < 0.0001; 57.2% vs. 19.1%, *p* < 0.0001) [60]. There was an increased incidence and severity of erosive esophagitis post-operatively. The study also newly diagnosed non-dysplastic Barrett’s esophagus postoperatively in 17.2% of patients [60]. A multi-center study of 90 patients, in which endoscopy prior to bariatric surgery was routinely performed and did not have evidence of Barrett’s esophagus preoperatively, noted a prevalence of Barrett’s esophagus of 18.8% in addition to increased prevalence of GERD symptoms, erosive esophagitis and PPI usage at a mean follow-up of 78 ± 15 months [61]. Barrett’s esophagus was significantly associated with weight loss failure in this study [61]. Similarly, Qumseya et al. performed a meta-analysis of 10 studies, including 680 patients who had undergone EGD from 6 months to 10 years after SG, and the pooled prevalence of BE was 11.6% (95% confidence interval [CI], 8.1–16.4%; *p* < 0.001; I2 = 28.7%) [62]. Most cases were noted after 3 years of follow-up and there was no significant association of BE with GERD symptoms in this study [62]. A meta-analysis of 46 studies with 10,718 patients revealed a prevalence of BE of 8% following SG [63]. There was a lack of correlation between symptoms and pathology as well [63]. This data suggests that there is a need for endoscopic surveillance following SG. The current studies regarding Barrett’s esophagus following SG have some limitations as outlined by a statement by American Society for Metabolic and Bariatric Surgery (ASMBS) [64]. The ASMBS has suggested to offer screening for BE in SG patients ≥3 years post-SG regardless of the presence of GERD symptoms [64].

Of note, reflux symptoms both pre- and post-operatively do not always correlate with objective evidence of GERD. A study involving obese patients under consideration for bariatric surgery reported 12.3% of patients with low GERD Questionnaire scores < 8 had erosive esophagitis [47]. 

There have been several proposed pathophysiological mechanisms for the development of de novo GERD or exacerbation of existing GERD following SG (Table 5).

Gastric sleeve anatomy plays an important role in predisposing obese patients to GERD [65]. In general, the narrower the sleeve, the higher the intragastric pressure, which may increase reflux. Keidar et al. noted that a stenosis of the middle portion of the sleeve and the angular notch with upstream dilation was associated with higher rates of GERD following SG [66]. To reduce this risk, the use of a larger bougie size for sleeve calibration has been explored. When compared to a 32-French bougie, a 42-French bougie was associated with improvement in GERD symptoms in 80% of patients post-operatively [67]. However, data regarding the optimal bougie size for weight loss and decreased propensity of reflux has been mixed [68,69,70,71]. Regarding the ideal shape of the SG, it has been suggested that the sleeve should be a trapezoid, being widest at the antrum and narrowest at the cardia, to prevent sleeve stenosis and increased intragastric pressure [65]. The antrum should be preserved, as demonstrated by Garay et al., as it may accelerate gastric emptying, reduce intragastric pressure and subsequent reflux [72]. Thus, the placement of the first staple line has been proposed to be >5 cm from the pylorus [72].

Caution must be exercised to avoid disruption of the natural anatomy of the esophagogastric junction, namely the gastroesophageal flap valve, the angle of His and gastric sling fibers, which are protective against reflux. The last staple line should not be placed too close to the esophagus, to avoid mechanical injury of the sling fibers and angle of His, nor too far from the esophagus, resulting in accessory gastric tissue retention [65]. Concomitant hiatal hernia should be repaired to prevent functional impairment of the esophagogastric junction [40]. It has been suggested that impaired esophagogastric motility may contribute to postoperative GERD though alterations in motility are often induced as part of the mechanism of the surgery itself [73]. A dynamic contrast study may also reveal esophageal dysmotility as a possible etiology of reflux and, thus, some experts recommend these interventions in patients with post-SG GERD [74]. Cephalad migration of the Z-line has also been implicated in post-operative GERD, and can be detected on endoscopy or contrast studies [38,60].

### 3.4. Management and Treatment Approach to GERD Following Bariatric Surgery

Following bariatric surgery, lifestyle modifications are recommended to include gradual advancing of the diet from liquids to eventually smaller portions of healthy, protein-based food. Exercise, nutritional supplementation, management of underlying constipation and avoidance of factors that exacerbate reflux, i.e., alcohol, tobacco, caffeine, chocolate and postprandial supination are also emphasized. Overfilling of the stomach with large meal portions may lead to regurgitation or reflux-like symptoms. After exclusion of anatomic and functional etiologies, dietary education, counseling and training should be provided to these patients.

Patients often receive PPI prophylaxis for weeks to months to prevent marginal ulcers and reflux symptoms. A meta-analysis of seven studies revealed an OR of marginal ulcer formation in the PPI group versus the non-PPI group to be 0.50 with low heterogeneity, indicating the PPI group had two times less ulceration compared to the non-PPI group [75]. In patients with GERD following bariatric surgery, PPI and histamine-2 receptor antagonists (H2RA) regimens should be optimized following bariatric surgery. There has been emerging data regarding the efficacy of the novel potassium-competitive acid blocker, vonoprazan, in improving GERD symptoms refractory to PPI therapy [76,77,78,79,80]. However, its use in GERD following bariatric surgery has not been evaluated thus far.

Given the robust evidence linking SG to an increased risk of GERD, the authors recommend a discussion of the potential exacerbation of pre-existing GERD or an increased risk of developing new-onset GERD during the shared decision-making process for bariatric surgery selection. According to a multi-society consensus statement from the ASMBS, the Society of American Gastrointestinal and Endoscopic Surgeons (SAGES), the American Society for Gastrointestinal Endoscopy (ASGE), the European Association of Endoscopic Surgery (EAES), the Society for Surgery of the Alimentary Tract (SSAT) and the Society of Thoracic Surgeons (STS), sleeve gastrectomy should not be performed as an anti-reflux procedure [81]. Patients with BMI > 35 and medically refractory GERD should be considered for either RYGB or fundoplication [81]. There are data to suggest an increased risk of hiatal hernia and GERD recurrence with fundoplication in patients with BMI > 35, and these patients may benefit from RYGB due to the additional weight loss benefit [81,82,83,84,85].

Post-surgical complications, including stenosis, kinking, angulation and marginal ulcers, must be recognized and treated appropriately. Stenosis, kinking and angulation may lead to postoperative GERD and obstructive symptoms due to increased intragastric pressure and reflux of gastric contents [86,87,88].

Endoscopic balloon dilation is a safe, effective and minimally invasive method for the management of gastric sleeve stenosis [89,90,91]. The optimal type and size of the balloon, including controlled radial expansion balloon and pneumatic achalasia balloons, and the number of dilations required are unclear [92].

Endoscopic balloon dilation has been demonstrated to be safe and effective for gastrojejunal strictures post-RYGB [93]. RYGB-associated strictures may also be safely treated with lumen-apposing metal stents (LAMS) [94,95,96]. In a prospective study of 412 patients who underwent RYGB, 14 patients developed a stricture, of which 12 had resolution of their stricture following LAMS insertion [94]. Two patients required surgery for refractory strictures that were associated with marginal ulceration of the gastrojejunostomy. A stent migration rate of 19% was noted, though stent migration did not seem to result in adverse patient outcomes in the study [94].

**Figure 2:**
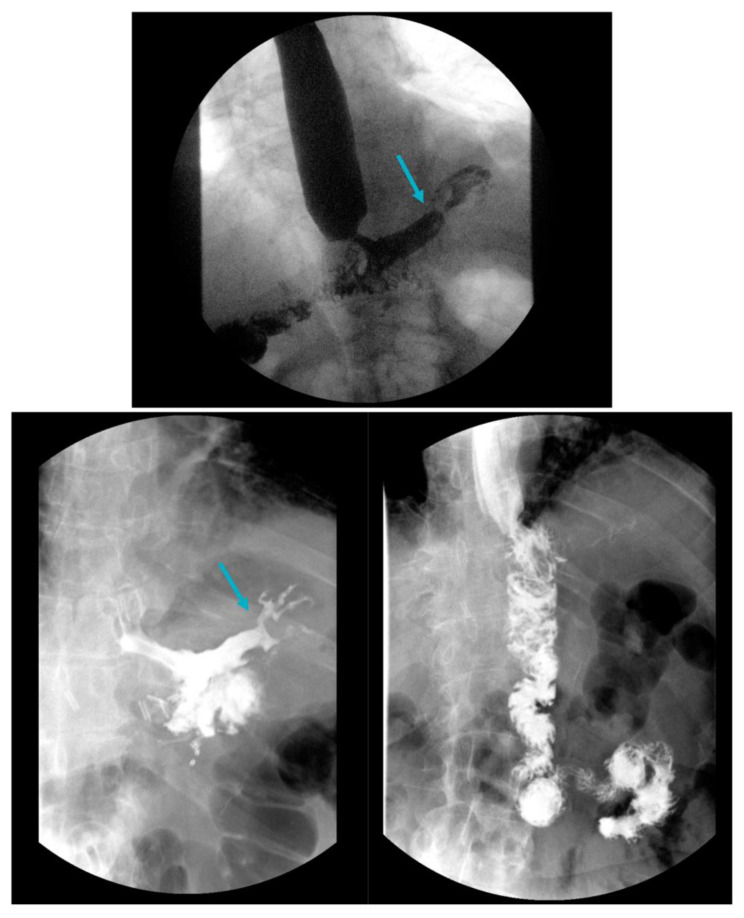
Upper gastrointestinal series demonstrating a Roux-en-Y gastric bypass with a gastrogastric fistula and leak under the left hemidiaphragm (arrows, **top** and **bottom left**) as evidenced by contrast filling of an irregularly shaped cavity lateral to the gastric pouch, which spontaneously empties back via the gastric pouch. This patient eventually required a distal esophagectomy, total gastrectomy and esophagojejunostomy (**bottom right**).

**Figure 3:**
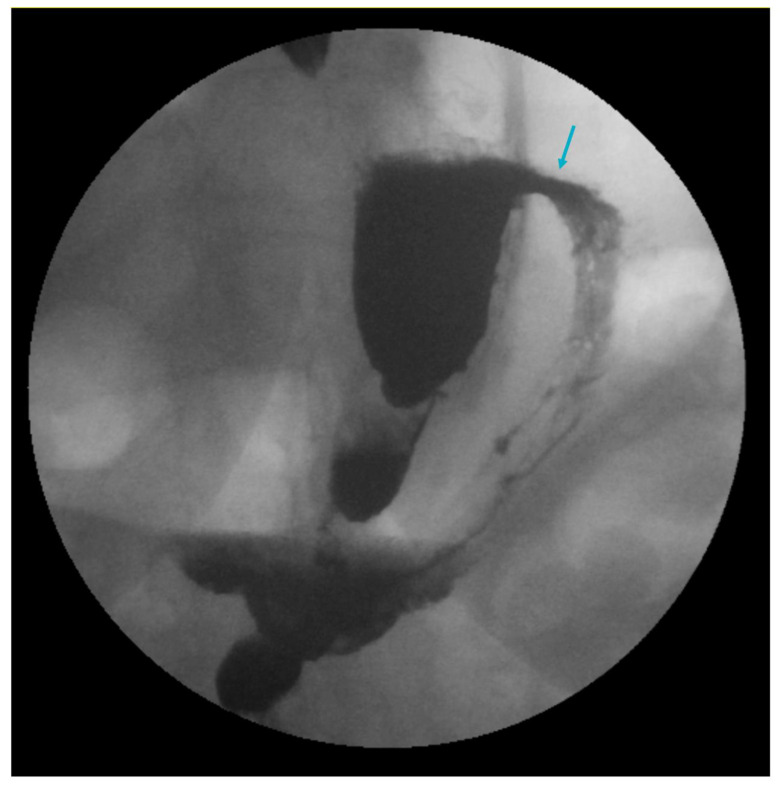
A gastrogastric fistula (arrow) in a patient with a prior Roux-en-Y gastric bypass as demonstrated on upper gastrointestinal series.

**Figure 4:**
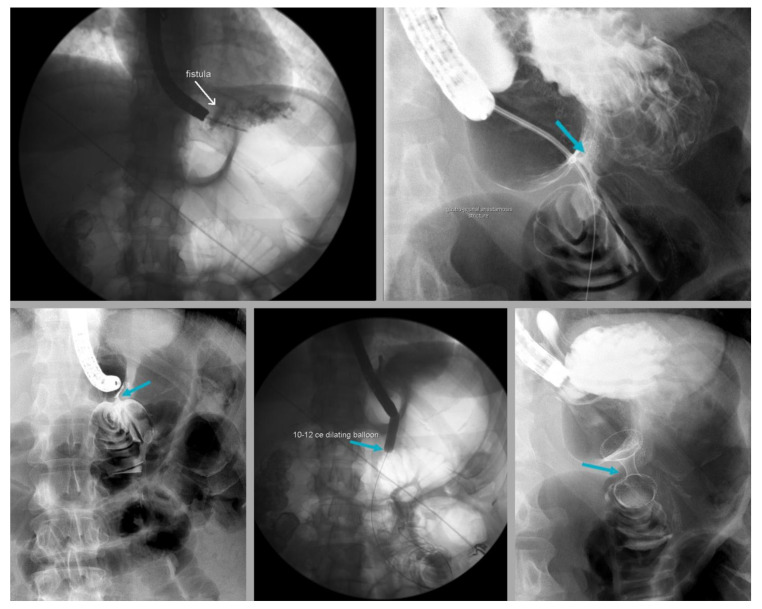
Upper gastrointestinal series in a patient with severe gastroesophageal reflux disease following Roux-en-Y gastric bypass, gastrogastric fistula (arrow, **top left**), gastrojejunal stricture (arrow, **top right** and arrow, **bottom left**), who underwent controlled radial expansion balloon dilation (arrow, **bottom middle**) and placement of lumen-apposing metal stent (arrow, **bottom right**).

**Figure 5:**
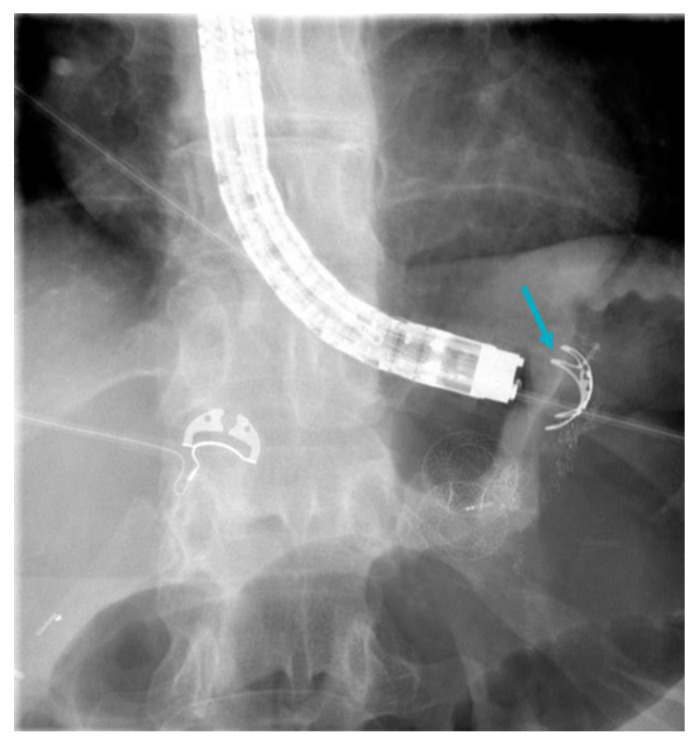
Persistent gastrogastric fistula despite suturing following Roux-en-Y gastric bypass which required endoscopic fistula closure utilizing an over-the-scope clip system “Bear Claw” Ovesco Endoscopy, Tübingen, Germany (arrow).

Gastrogastric (GG) fistulas are a serious complication that can occur in up to 6% of RYGB cases, and are often associated with abdominal pain, GERD and weight gain [97,98]. GERD in GG fistula has been attributed to the passage of gastric acid from the remnant stomach to the gastric pouch through the fistula (Figure 2 and Figure 3). Endoscopic repair offers a minimally invasive method to the treatment of GG fistulas (Figure 4 and Figure 5) [99]. Various endoscopic techniques have been utilized for endoscopic closure of GG fistulas, including over-the-scope clips, through-the-scope clips and endoscopic suturing [100,101,102,103,104]. The best outcomes for endoscopic repair of GG fistulas tend to occur with small fistulas that are <10 mm in size [105]. In a study of 95 patients, initial complete closure of GG fistula was successful in 95% of patients, though 65% had reopening [105]. There were no GG fistulas > 20 mm in size that remained closed during the follow-up period, compared to 32% of GG fistulas ≤ 10 mm in size that remained closed [105]. In another study of 99 RYGB patients with GGF, endoscopic GGF and surgical revision had similar rates of GERD resolution [106]. Endoscopic GGF was associated with a greater improvement in abdominal pain and less adverse events compared to surgical revision, which resulted in a greater weight loss [106]. Ultimately, surgical revision may be required to treat GG fistulas, especially with large defects [107].

Post-operative leaks following bariatric surgery are a serious complication that can often result in significant morbidity and mortality. The use of endoscopic self-expandable metal stents has shown to be effective in the management of staple-line leaks following bariatric surgery [100,108]. Endoscopic internal drainage, in conjunction with perigastric abscess drainage, have also been shown to be promising [109].

**Figure 6:**
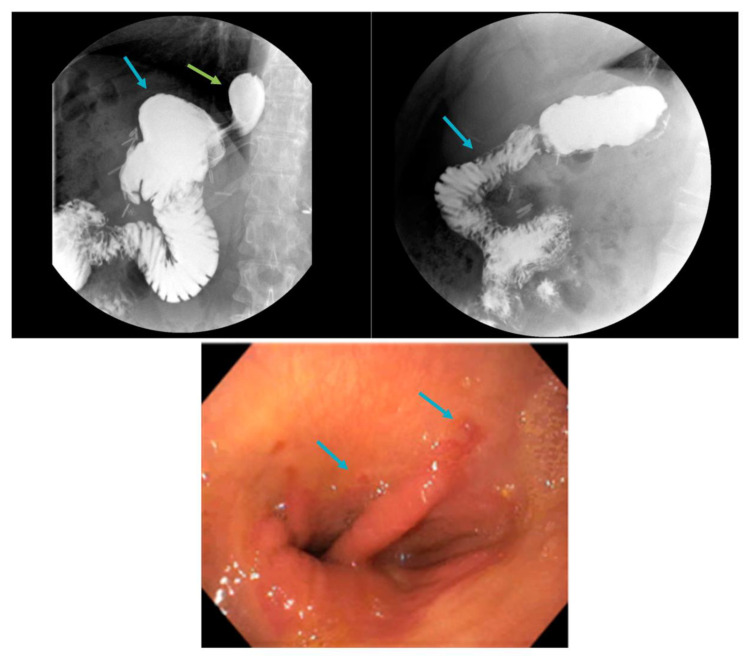
Large gastric pouch (blue arrow, **top left**) with sliding hiatal hernia (green arrow, **top left**) and a short, 20 cm Roux limb (**top right**) seen on upper gastrointestinal series, in addition to Los Angeles Grade A esophagitis on upper endoscopy (arrows, **bottom**).

It is important to note that the length of the Roux-en-Y limb, on average 120 cm, and the size of the gastric pouch are both critical to decrease the risk of complications such as GERD (Figure 6). It has been demonstrated that the optimal gastric pouch should be 20 to 30 cc in volume and primarily involve the lesser curvature of the stomach [110]. Revisional surgery may be needed to optimize the pouch size. A systematic review of 13 studies suggested that the length of the common limb >400 cm confers weight loss and metabolic benefits [111]. With regard to SG, surgical techniques and anatomy of the sleeve should be optimized as described in the section above to prevent post-SG GERD. If the sleeve gastrectomy has been performed correctly, an anti-reflux procedure should not be indicated in most cases.

Recently, endoscopic sleeve gastroplasty (ESG), also known as endoluminal vertical gastroplasty (EVG), has shown to be a safe and effective method for weight loss in the setting of weight recidivism post-LSG, but studies that have evaluated the effects of ESG on GERD are limited [112,113,114,115,116]. Fayad et al. revealed that new-onset GERD was notably lower in the ESG group than in the LSG group (1.9% vs. 14.5%, *p* < 0.05), due to sparing of the fundus of the stomach [117]. A study of 64 patients revealed no incidence of reflux symptoms at 1 year following EVG [118]. In the Transoral Gastric Volume Reduction as Intervention for Weight Management (TRIM) trial, which explored the safety and efficacy of transoral gastric volume reduction surgery using an endoscopic suturing system, 8 of 14 patients reported reflux symptoms prior to the procedure, and 5 out of 14 at 1-year follow-up [119].

Sleeve gastrectomy has also been combined with fundoplication in an attempt to minimize postoperative GERD. In a randomized clinical study of 278 patients comparing SG to SG with Rossetti fundoplication (RF) in terms of de novo GERD, there was a significant reduction in PPI use (4.3% vs. 17.1%) and esophagitis (2% vs. 23.4) in the SG with RF group compared to the SG group [120]. Olmi et al. described 220 obese patients who underwent LSG and modified RF with a 24-month follow-up. It was noted that 98.5% of cases did not report GERD or use PPIs postoperatively, and approximately 97% of cases had an improvement in esophagitis on endoscopy [121]. Uccelli et al. reported 127 patients who underwent sleeve gastrectomy with RF, of which 74.8% had GERD prior to the surgery and 95% did not have reflux symptoms at 5-year follow-up. Improvement in esophagitis on endoscopy was also noted, though the study had some limitations [122,123]. A significant increase in the lower esophageal sphincter tone was demonstrated on high-resolution esophageal manometry in 20 patients following LSG with RF, thereby potentially reducing the likelihood of developing long-term GERD [124]. Nocca et al. conducted a prospective study of 25 patients who underwent laparoscopic Nissen-sleeve gastrectomy, among which the majority had GERD symptoms, and 76% of patients remained asymptomatic without PPI use three months postoperatively [125]. Similar results in GERD improvement were noted in several other studies [126,127,128,129,130,131,132].

A systematic review and meta-analysis of 15 studies explored the efficacy of SG with hiatal hernia repair versus SG with fundoplication and concluded that, while both procedures were effective in GERD resolution and weight loss, SG with fundoplication was superior in terms of GERD resolution, though had a higher complication rate [133]. Carandina et al. reviewed the safety of combined sleeve gastrectomy with fundoplication in a review of seven studies with 487 patients, and revealed a postoperative complication rate of 9.4% [134]. Gastric perforation, bleeding and sleeve stenosis were the most common reported complications [134]. Further studies are warranted to determine the role of SG with fundoplication. Though SG with fundoplication seems to be a safe surgical procedure with an acceptable rate of early postoperative complications, the authors currently feel that there is a lack of high-quality, long-term data regarding the safety, efficacy and technical feasibility for SG with fundoplication to suggest this approach [133,134].

Ultimately, many patients require conversion from LSG to RYGB, which is highly effective in the treatment of post-bariatric surgery GERD (Table 6). The literature has revealed a 5–10% conversion rate from LSG to RYGB due to GERD [59,135,136,137]. Parmar et al. reviewed 22 conversions from LSG to LRYGB, among which 10 were for intractable GERD. Improvement in GERD was noted in all patients and 80% of patients discontinued acid suppression medications [138]. Similarly, Abdemur et al. reported complete resolution of GERD after LSG to LRYGB conversion in six out of nine patients [139]. Hendricks et al. reported four conversions from LSG to LRYGB, of which three had complete resolution of their symptoms and one patient had partial resolution [140]. Similar results were obtained by several other studies [141,142,143,144,145].

**Figure 7:**
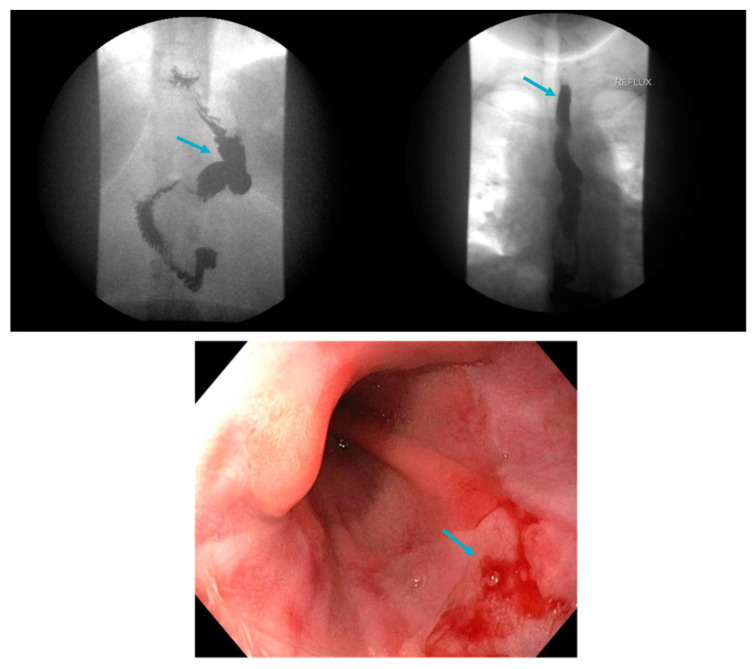
Sliding hiatal hernia (arrow, **top left**) and reflux (arrow, **top right**) demonstrated on upper gastrointestinal series in addition to severe erosive esophagitis on upper endoscopy (arrow, **below**) in a patient following sleeve gastrectomy.

In patients with medically refractory GERD following sleeve gastrectomy, the authors suggest obtaining an esophagram and selective esophagogastroduodenoscopy, manometry and pH testing to further assess the anatomy and physiology of the symptoms. It is important to determine the presence of a hiatal hernia, as this should be corrected at the time of conversion to RYGB (Figure 7). A small subset of surgeons have performed the Hill procedure using the phrenoesophageal bundle for persistent GERD following RYGB [146]. The widespread application of the Hill procedure for this purpose has been limited due to surgeon familiarity and expertise.

As discussed above, some studies have proposed endoscopic surveillance post-bariatric surgery to assess disease progression to Barrett’s esophagus and esophageal adenocarcinoma [147,148,149]. Endoscopy prior to bariatric surgery and obtaining a thorough history regarding gastroesophageal reflux symptoms may improve patient selection for bariatric surgery [135]. Carabotti et al. demonstrated that the incidence of endoscopic lesions was the same in patients who reported reflux symptoms and those who did not, and concluded that symptoms cannot be reliably considered as an indication for endoscopy in these patients [150]. The ASMBS has suggested to offer screening for BE in SG patients ≥3 years post-SG, regardless of the presence of GERD symptoms, in addition to the standard screening indications for GERD and Barrett’s esophagus [64].

Finally, the use of magnetic sphincter augmentation devices, such as the LINX© system following SG, has shown some promising results, though the data are limited to small, retrospective studies. Rausa et al. performed a meta-analysis of three retrospective studies with 33 patients who underwent magnetic sphincter augmentation (MSA) for GERD following bariatric surgery. The pooled mean difference in preoperative GERD-Health-Related Quality of Life Questionnaire (GERD-HRQL) score was 17.5, which was statistically significant [151]. In a multicenter, single-arm prospective study of 30 patients over 12 months, MSA following LSG revealed improved GERD symptoms, reduced PPI use and decreased distal acid exposure in a minimally invasive manner [152]. A retrospective review of 13 identified patients who underwent LINX placement after bariatric surgery also noted decreased PPI usage post-operatively, reduced GERD-HRQL scores and relative safety of the procedure [153]. Additional studies have corroborated these findings [136,137,138,154,155,156]. Further data are needed to establish the safety and efficacy of magnetic sphincter augmentation devices following bariatric surgery.

## 4. Conclusions

GERD is a common, chronic condition which can significantly impact quality of life and lead to serious complications. Obesity is a well-established risk factor for GERD, which often improves with weight loss and bariatric surgery. With the recent rise in bariatric surgery, especially SG, persistent and de novo reflux following bariatric surgery has become a topic of concern. Management of post-bariatric surgery GERD includes lifestyle modifications, optimization of PPI and H2RAs, treatment of postoperative complications and repair of hiatal hernia if present. Conversion to Roux-en-Y currently has the most robust evidence to support its safety and efficacy for the treatment of medically refractory GERD post-SG. Other options include magnetic sphincter augmentation, though data regarding safety and efficacy are limited. Currently and going forward, more precise, standardized methods are warranted to document GERD following bariatric surgery, due to the variability in the reported literature. As bariatric surgery can be both the cure and the cause for GERD in the morbidly obese population, careful patient selection and proper surgical technique are paramount for a favorable outcome.

## Figures and Tables

**Table 1:** Mechanical factors that have been suggested to predispose patients with obesity to gastroesophageal reflux disease.

Pathophysiologic Mechanisms That Predispose Obese Patients to Reflux
Transient lower esophageal sphincter relaxations
Increased intra-abdominal pressure
Augmented gastroesophageal pressure gradient
Increased prevalence of hiatal hernia

**Table 2:** An overview of the literature pertaining to the remission of gastroesophageal reflux symptoms and the use of acid suppression medications pre- and post-Roux-en-Y gastric bypass. GERD = gastroesophageal reflux disease, PPI = proton pump inhibitors, RYGB = Roux-en-Y gastric bypass.

Remission of Gastroesophageal Reflux Symptoms and Use of Acid Suppression Medication Pre- and Post-Roux-en-Y Gastric Bypass						
Author	Year	Journal	Article Type	Number of Cases/Studies	GERD Symptom Remission	Pre- and Post-RYGB Usage of Acid Suppression Medications
Frezza et al. [51]	2002	Surgical Endoscopy	Prospective study	152	Heartburn 87% → 22%, *p* < 0.001 Water brash 18% → 7%, *p* < 0.05 Wheezing 40% → 5%, *p* < 0.001 Laryngitis 17% → 7%, *p* < 0.05 Aspiration 14% → 2%, *p* < 0.01	PPI: pre-RYGB 44% → post-RYGB 9%, *p* < 0.001 Histamine-2 receptor antagonists: pre-RYGB 60% → post-RYGB 10%, *p* < 0.01
Perry et al. [52]	2004	Journal of Society of Laparoendoscopic surgeons	Prospective study	57	All patients reported improvement or no symptoms of GERD after RYGB	PPI: 31 patients pre-RYGB → 3 patients post-RYGB High-dose histamine-2 receptor antagonists: 17 patients pre-RYGB → all patients treated with ranitidine 150 mg per day post-RYGB

**Table 3:** Literature comparison of sleeve gastrectomy and Roux-en-Y gastric bypass in terms of remission of gastroesophageal reflux symptoms and the use of acid suppression medications. ACM = acid suppression medications, EE = erosive esophagitis, GERD = gastroesophageal reflux disease, OR = odds ratio, PPI = proton pump inhibitors, (L)RYGB = (laparoscopic) Roux-en-Y gastric bypass, (L)SG = (laparoscopic) sleeve gastrectomy.

Comparison of Sleeve Gastrectomy and Roux-en-Y Gastric Bypass in Terms of Gastroesophageal Reflux Symptom Remission and the Use of Acid Suppression Medications									
Author	Year	Journal	Article Type	Number of Cases/Studies	GERD Symptom Remission—RYGB	Pre- and Post-Operative Usage of Acid Suppression Medications (ACM)	GERD Symptom Remission—SG	*p*-Value	Additional Comments
Peterli et al. [50]	2018	JAMA	Randomized controlled trial	217	60.4%	N/R	25%	0.002	De novo reflux in 31.6% after SG vs. 10.7% after RYGB (*p* = 0.01)
Alghamdi et al. [53]	2022	Frontiers in Surgery	Systematic review, meta-analysis	16	Odds ratio of GERD remission = 3.16 for LRYGB compared to LSG, *p* = 0.003, heterogeneity N/A Usage of ACM was not reported				There was no significant statistical difference between LRYGB and LSG with regard to new-onset GERD; heterogeneity was noted
Gu et al. [11]	2019	Obesity Surgery	Systematic review, meta-analysis	23	OR for GERD after LSG compared to LRYGB = 5.10, *p* < 0.001 LRYGB had a better effect on GERD compared to LSG, OR = 0.19, *p* < 0.001				
DuPree et al. [16]	2014	JAMA Surgery	Retrospective review	4832	62.8%	N/A	15.9%	*p* < 0.001	New-onset GERD was noted in 8.6% in the LSG group
Sheppard et al. [12]	2015	Obesity Surgery	Retrospective review	387	Pre-operative PPI use in LSG: 28% → 2% were able to discontinue PPI after SG Pre-operative PPI use in LRGYB: 32% → 33% were able to discontinue PPI after RYGB				
Matar et al. [15]	2020	Obesity Surgery	Retrospective review	517	EE prevalence higher after SG than RYGB (37.9% vs. 17.6%, *p* = 0.0001)				

**Table 4:** An overview of the literature pertaining to the remission of gastroesophageal reflux symptoms and the use of acid suppression medications pre- and post-sleeve gastrectomy. ACM = acid suppression medication, GERD = gastroesophageal reflux disease, (L)SG = (laparoscopic) sleeve gastrecromy, N/R = not reported.

Exacerbation or Remission of Gastroesophageal Reflux Symptoms and the Use of Acid Suppression Medications Pre- and Post-Sleeve Gastrectomy							
Author	Year	Journal	Article Type	Number of Cases/Studies	GERD Symptom Remission	Pre- and Post-SG Usage of Acid Suppression Medications	Additional Comments
Carter et al. [17]	2011	Surgery for Obesity and Related Diseases	Retrospective review	176	Pre-SG, 34.6% had GERD symptoms Post-SG, 49% reported GERD symptoms within 30 days 47.2% had persistent GERD (>1 month after LSG)	Pre-LSG: 22% → Post-LSG 33.8% of patients on GERD medication, *p* = 0.0428	
Tai et al. [13]	2013	Surgical Endoscopy	Retrospective review	66	Prevalence of GERD symptoms pre-LSG 12.1% → 47% post-LSG, *p* < 0.001	N/R	New-onset GERD after LSG: 44.8% Hiatal hernia: 6.1% → 27.3%
Howard et al. [14]	2011	Surgery for Obesity and Related Diseases	Retrospective review	28	25% pre-LSG → 23% post-LSG, *p* < 0.05	Pre-LSG: 25% → post-LSG 39% with GERD symptoms despite ACM use	New-onset GERD post-LSG: 22%

**Table 5:** Proposed mechanisms for de novo or increased gastroesophageal reflux following bariatric surgery.

Proposed Mechanisms for De Novo or Increased Gastroesophageal Reflux following Bariatric Surgery	
Sleeve Gastrectomy	Roux-en-Y Gastric Bypass
Loss of the antireflux barrier ○ Disruption of the esophagogastric junction, gastroesophageal flap valve, the angle of His, gastric sling fibers, fundal resection Functional impairment of the gastroesophageal junction ○ Baseline hiatal hernia Increased intragastric pressure ○ Narrow sleeve dimensions ○ Sleeve stenosis, angulation or kinking ○ Incorporation of the antrum into the sleeve resection ○ Overfilling of sleeve due to a large meal size Sleeve leak	Functional impairment of the gastroesophageal junction ○ Baseline hiatal hernia Anastomotic stenosis Large gastric remnant Gastrogastric fistula

**Table 6:** An overview of the literature on the effect of conversion from sleeve gastrectomy to Roux-en-Y gastric bypass on gastroesophageal reflux disease. ACM = acid suppression medication, GERD = gastroesophageal reflux disease, RYGB = Roux-en-Y gastric bypass, N/R = not reported.

The Effect of Conversion from Sleeve Gastrectomy to Roux-en-Y Gastric Bypass on Gastroesophageal Reflux Disease					
Author	Year	Journal	Article Type	Conversion Rate to RYGB for GERD (%)	Effect on GERD Symptoms and Use of Acid Suppression Medications
Langer et al. [136]	2010	Obesity Surgery	Retrospective review	11	100% of patients with severe reflux discontinued ACM
Salminen et al. [137]	2018	JAMA	Randomized clinical trial	6	N/R
Parmar et al. [138]	2017	Obesity Surgery	Prospective study	45	100% of patients reported improvement in GERD symptoms 80% of patients were able to discontinue ACM
Abdemur et al. [139]	2016	Surgery for Obesity and Related Diseases	Retrospective review	0.8	66% of patients had complete resolution of GERD symptoms
Hendricks et al. [140]	2016	Surgery for Obesity and Related Diseases	Retrospective review, comparative study	10.5	75% of patients had complete resolution of GERD symptoms 25% of patients had partial resolution
Gautier et al. [141]	2013	Obesity Surgery	Retrospective review	33.3	100% of patients discontinued ACM No recurrence of GERD was noted
Strauss et al. [142]	2023	Surgical Endoscopy	Retrospective review	72.2	80.2% of patients had improvement in GERD symptoms 19.4% of patients were able to discontinue ACM
Felsenreich et al. [144]	2022	Obesity Surgery	Retrospective review	34.2	29.9% of patients reported GERD symptoms following conversion
Peng et al. [145]	2020	Surgery for Obesity and Related Diseases	Systematic Review and Meta-analysis	N/R	57.1–100% had remission or improvement in GERD symptoms

## Data Availability

Not applicable.
